# Changes in the Cerebrospinal Fluid and Plasma Lipidome in Patients with Rett Syndrome

**DOI:** 10.3390/metabo12040291

**Published:** 2022-03-25

**Authors:** Martina Zandl-Lang, Thomas Züllig, Martin Trötzmüller, Yvonne Naegelin, Lucia Abela, Bernd Wilken, Sabine Scholl-Buergi, Daniela Karall, Ludwig Kappos, Harald Köfeler, Barbara Plecko

**Affiliations:** 1 Department of Paediatrics and Adolescent Medicine, Division of General Paediatrics, University Childrens’ Hospital Graz, Medical University of Graz, 8036 Graz, Austria; martina.zandl@medunigraz.at (M.Z.-L.); barbara.plecko@medunigraz.at (B.P.); 2 Institute of Molecular Biosciences, NAWI Graz, University of Graz, 8010 Graz, Austria; thomas.zuellig@medunigraz.at; 3 Core Facility Mass Spectrometry, Medical University of Graz, 8010 Graz, Austria; martin.troetzmueller@medunigraz.at; 4 Neurologic Clinic and Policlinic, Departments of Medicine, Biomedicine, and Clinical Research, University Hospital Basel, University of Basel, 4031 Basel, Switzerland; yvonne.naegelin@usb.ch (Y.N.); ludwig.kappos@usb.ch (L.K.); 5 Department of Child Neurology, University Children’s Hospital Zurich, 8032 Zurich, Switzerland; lucia.abela@kispi.uzh.ch; 6 Department of Pediatric Neurology, Klinikum Kassel, 34125 Kassel, Germany; bernd.wilken@gnh.net; 7 Clinic for Paediatrics I, Inherited Metabolic Disorders, Medical, University of Innsbruck, 6020 Innsbruck, Austria; sabine.scholl-buergi@tirol-kliniken.at (S.S.-B.); daniela.karall@i-med.ac.at (D.K.)

**Keywords:** rare diseases, Rett syndrome, LC-MS, metabolomics, lipidomics, biomarker

## Abstract

Rett syndrome (RTT) is defined as a rare disease caused by mutations of the methyl-CpG binding protein 2 (MECP2). It is one of the most common causes of genetic mental retardation in girls, characterized by normal early psychomotor development, followed by severe neurologic regression. Hitherto, RTT lacks a specific biomarker, but altered lipid homeostasis has been found in RTT model mice as well as in RTT patients. We performed LC-MS/MS lipidomics analysis to investigate the cerebrospinal fluid (CSF) and plasma composition of patients with RTT for biochemical variations compared to healthy controls. In all seven RTT patients, we found decreased CSF cholesterol levels compared to age-matched controls (*n* = 13), whereas plasma cholesterol levels were within the normal range in all 13 RTT patients compared to 18 controls. Levels of phospholipid (PL) and sphingomyelin (SM) species were decreased in CSF of RTT patients, whereas the lipidomics profile of plasma samples was unaltered in RTT patients compared to healthy controls. This study shows that the CSF lipidomics profile is altered in RTT, which is the basis for future (functional) studies to validate selected lipid species as CSF biomarkers for RTT.

## 1. Introduction

Rett syndrome (OMIM 312750, RTT) is defined as an orphan disease with a prevalence of 1:10,000 to 1:15,000 live female births [1]. It is one of the most common causes of genetic mental retardation in girls caused by loss-of-function mutations of the methyl-CpG-binding protein 2 (*MECP2*) located on chromosome X [2]. While MeCP2 is ubiquitously expressed, it is particularly abundant in the brain [3]. Its main function is to bind to methylated DNA and coordinate gene expression through activation and repression, although its role in chromatin remodeling and mRNA splicing has also been described [4,5]. In the central nervous system (CNS) MeCP2 is primarily expressed in neurons and to a lesser extent also in glial cells. It correlates with postnatal maturation and neuronal differentiation, illustrating its importance in CNS function and maintenance [6]. 

Symptoms in RTT progress over time and appear in four progressive stages. Following a period of seemingly normal infantile psychomotor development which lasts for 6–18 months after birth, the disease is characterized by a phase of stagnation with developmental progress delay (e.g., sitting, crawling, talking). In the stage of rapid regression acquired motor and mental skills are lost and breathing abnormalities and seizures occur. These stages are followed by a plateau stage and a stage of motor deterioration. Epilepsy occurs in 60–80% of all cases with RTT whereas incidences are even higher in early onset RTT and in RTT with more severe developmental disabilities [7]. Due to cardiac involvement and respiratory compromisation, RTT is associated with a relatively high rate of premature deaths ranging from 1.2% to 3.9%, with 26% of these patients experiencing a sudden unexplained death [8,9]. RTT is also characterized by metabolic perturbations, such as elevated oxidative stress [10], mitochondrial dysfunction [11], but also dyslipidemia [12]. 

Previous studies in RTT model mice reported an altered cholesterol metabolism similar to RTT patients [13,14,15]. *Mecp2*-null mice display perturbed lipid homeostasis, including high serum triglyceride and cholesterol levels, and elevated cholesterol in whole brain homogenates. Further, Buchovecky et al. showed that nonsense mutations in Squalene monooxygenase, one of the rate-limiting enzymes in cholesterol biosynthesis, as well as statin treatment rescue a disrupted lipid metabolism and improve symptoms in *Mecp2*-null mice [4]. In humans, metabolic parameters vary heavily within the RTT population. Nevertheless, analysis of serum samples of RTT patients revealed abnormal lipid parameters, such as elevated cholesterol, triglycerides and LDL levels [16]. Additionally, scavenger receptor B class 1 (SR-B1), a protein responsible for the uptake of cholesteryl esters from HDL and LDL particles, was found to be reduced in RTT patient fibroblasts, suggesting a link between lipid homeostasis and MeCP2 function [17]. To date, RTT lacks a specific biomarker and underlying pathogenic mechanisms of RTT are far from being understood. New treatment options are under investigation, but current treatment is restricted to symptom control.

Multi-omics approaches, including Next Generation Sequencing (NGS) and mass spectrometrical (MS) analysis, offer enormous potential for in-depth analysis of rare diseases. The investigation of disease-associated pathomechanisms is especially important for the development of treatment options but can also be useful in case NGS reveals variants of unknown significance (VUS). To date, 215 *MECP2* variants are classified as VUS [18]. Sophisticated algorithms are developed to increase prediction rates for the pathogenicity of variants [19]. Deep biochemical phenotyping applying proteomics, metabolomics and/or lipidomics performed by MS may help to underpin functional impairment of the MeCP protein and thus prove pathogenicity of variants that have hitherto been classified as VUS. 

Recent advances in MS, bioinformatics tools and software enable huge diagnostic progress through an unbiased, comprehensive and rapid analysis of the global metabolic content of a distinct biological sample. The metabolome represents the global spectrum of low molecular-weight (<1 kDa) metabolites and provides a snapshot of its biochemical composition in the respective body fluid (5). As such, alterations of the metabolome reflect genomic, transcriptomic and proteomic changes and can accelerate the diagnosis of neurometabolic disorders [20]. Lipidomics profiling, a subset of metabolomics devoted to the qualitative and quantitative analysis of lipids, has emerged as a promising new tool for identifying alterations in lipid classes and finding new biomarker candidates [14,15,21,22]. To date, blood plasma is the most common material for clinical diagnostic analysis due to minimally invasive collection and its rich composition of metabolites. CSF on the other hand is more challenging to collect, but due to its proximity to the CNS, CSF may provide a more accurate and unique reflection of the neurochemical condition.

The aim of this study was to establish metabolomics/lipidomics profiles of CSF and plasma samples from RTT patients and healthy individuals in order to obtain detailed and deep insights into metabolic perturbation. 

## 2. Results

### 2.1. Demographic Information of Study Cohort and Quality Control

**Figure 1:**
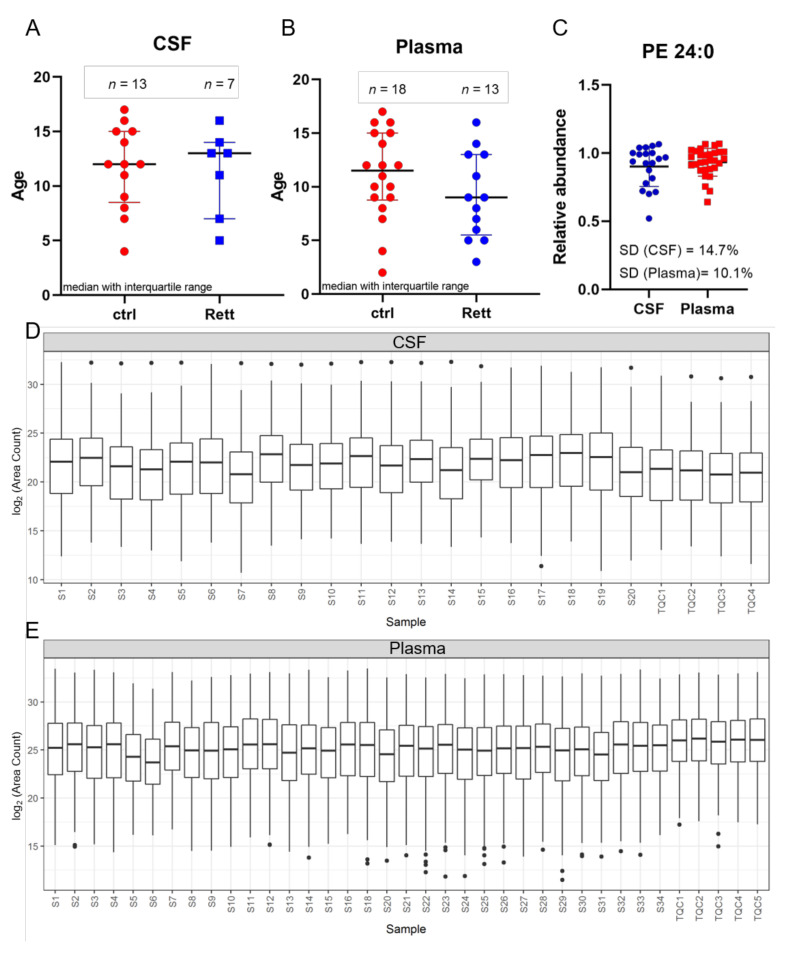
Age distribution and quality control. Demographic information and median age on the study subjects ((**A**) for CSF; (**B**) for plasma) (**C**) Phosphatidylethanolamine (PE) 24:0 as extraction control for sample preparation and UHPLC-MS/MS analysis. Standard deviation (SD) for CSF is 14.7% and for plasma samples 10.1%. (**D**,**E**) Overview of all CSF (**C**) and plasma (**D**) samples analyzed by MS shown as boxplot of all lipids and as sum of the total ion current to exclude inconsistencies in sample preparation and detection. Dots display single lipid species outliers of the whole lipidome.

In this study, CSF samples were taken from 13 female disease controls and seven female patients suffering from RTT with a median age of 12 years and 13 years, respectively (Figure 1A). Additionally, plasma samples were taken from a total of 18 female controls and 13 female RTT patients with a median age of 11.5 years and 9 years, respectively (Figure 1B). Phosphatidylethanolamine (PE) 24:0 was used as extraction control and added to each sample prior to analysis (Figure 1C). Standard deviation (SD) was found to be very low (14.7% for CSF samples and 10.1% for plasma samples) indicating unbiased sample preparation and analysis. All lipids detected in CSF and plasma samples were plotted in a boxplot reflecting the sum of the total ion current (TIC) and revealed no inconsistencies in the sample extraction or detection (Figure 1D,E). 

### 2.2. Multivariate Analysis (MVA) of RTT Patients Compared to Healthy Controls

Data obtained by UHPLC-MS/MS underwent metabolite and lipid identification with the Lipid Data Analyzer (LDA) software (version 2.8.0, Graz, Austria), followed by statistical univariate and multivariate analysis with the program *R* and the lipidr package [23]. In a pilot study, using CSF and plasma samples of six RTT patients obtained from the FINGORETT study we performed metabolomics and lipidomics analysis. In contrast to multivariate analysis (MVA) of the lipidome, MVA of metabolomics data showed no significant separation of the control versus the patient group in this rather small study cohort (data not shown). Therefore, we decided to focus our attention on lipidomics analysis for further analysis in a larger patient cohort. Additionally, oral treatment with the sphingosine-1-phosphate modulator Fingolimod had no impact on the metabolomics or lipidomics profile of RTT patients (data not shown). Of note, results published by Naegelin et al. in 2020 showed, that oral treatment with Fingolimod is safe, but reveals no efficacy on laboratory, clinical and imaging measures of RTT patients [24]. 

**Figure 2:**
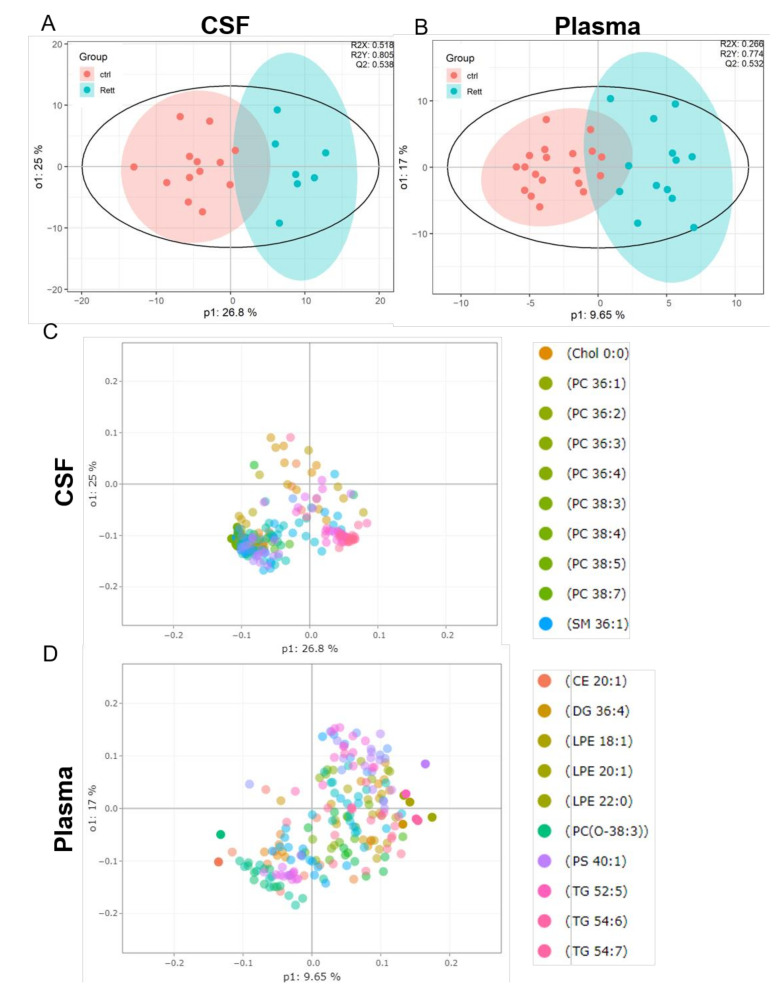
Supervised multivariate statistical analysis of the CSF (**A**,**C**) and plasma (**B**,**D**). (**A**,**B**) Orthogonal partial least square-discriminant analysis (OPLS-DA) plots were generated using the lipidome data set obtained from UHPLC-MS/MS analysis and the program R with the lipidr package. (**C**,**D**) Loading plot of CSF (**C**) and plasma (**D**) samples obtained from RTT patients compared to healthy controls. Scattered dots represent various lipid species that were identified as influential variables in the discriminant analysis. The top 10 variables are listed in the right panel. CE (Cholesterol Ester), Chol (Cholesterol), DG (Diacylglycerol), PC (Phosphatidylcholine), LPE (Lyso-Phosphatidylethanolamine), PS (Phosphatidylserine), SM (Sphingomyelin), TG (Triacylglycerol).

OPLS-DA analysis revealed a clear separation of the patient group (blue) versus the control group (red) in both, the CSF (Figure 2A) and plasma samples (Figure 2B). The evaluation parameters for the obtained OPLS-DA models were: R2X = 0.518; R2Y = 0.805; Q2Y = 0.538 in the CSF and R2X = 0.266; R2Y = 0.774; Q2Y = 0.532 in the plasma samples indicating good predictive power and well modeled metabolites. Loading plots including the top 10 variables obtained from OPLS-DA are shown in Figure 2C, D. The top 50 variables are listed in tables in the supplement (Tables S2 and S3). We could show that especially cholesterol, phosphatidylcholine (PC) species (36:1, 36:2, 36:3, 36:4, 38:3, 38:4, 38:5, 38:7) and sphingomyelin (SM 36:1) are significant variables in the CSF of RTT patients compared to healthy controls. In the plasma cholesterol ester (CE) 20:1, diacylglycerol (DG) 36:4, various phospholipid (PL) (lysophosphatidylethanolamine LPE18:1, LPE 20:1, LPE 22:0, ether-PL PC-O 38:3, phosphatidylserine PS 40:1) and triacylglycerol (TG 52:5, TG 54:6) species are major discriminators between RTT and control samples. PCA plot, permutation tests and observation diagnostics performed with CSF and plasma data are listed in Figure S1. 

### 2.3. Univariate Analysis (UVA) of RTT Patients Compared to Healthy Controls

Lipidomics data obtained from UHPLC-MS/MS analysis further underwent univariate analysis using Wilcoxon rank-sum test using FDR as multiple comparison correction and are depicted as boxplots. Results are presented as log_2_ values of total area counts and are additionally listed as Tables S3 and S4 of the supplement. 

**Figure 3:**
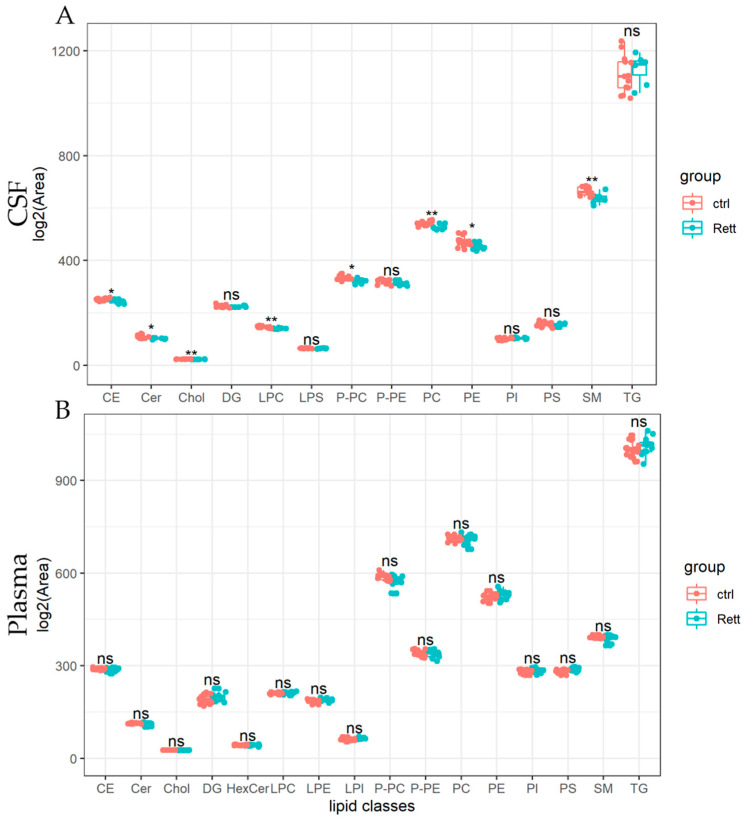
Univariate analysis of lipid classes analyzed from CSF and plasma samples of RTT patients compared to healthy controls. Lipidome data obtained from UHPLC-MS/MS were analyzed using R and the lipidr package. Data is shown as log^2^ of total area count. Box plots represent lipid classes detected in the CSF (**A**) and plasma (**B**) of RTT patients and controls and are calculated as sum of lipid species detected in one lipid class. CE (Cholesterol Ester), Cer (Ceramide), Chol (Cholesterol), DG (Diacylglycerol), HexCer (Hexosylceramide), PC (Phosphatidylcholine), PE (Phosphatidyl-ethanolamine), PI (Phosphatidylinositol), PS (Phosphatidylserine), LPC (Lyso-PC), LPE (Lyso-PE), LPI (Lyso-PI), PC-O (Ether-linked PC), PE-P (Plasmalogens of PE), SM (Sphingomyelin), TG (Tri-acylglycerol); *—FDR adjusted *p* < 0.05 by Wilcoxon rank-sum test, **—FDR adjusted *p* < 0.01 by Wilcoxon rank-sum test, ns-not significant.

Lipids detected in the CSF and plasma of RTT patients and controls are presented as lipid classes and lipid species. Results are shown as lipid classes (=sum of lipid species detected in one lipid class) should provide an overview of lipids detected in CSF and plasma samples and changes in the lipid distribution. We identified 14 lipid classes in the CSF (Figure 3A) and 16 lipid classes in the plasma (Figure 3B). Levels of cholesterol (Chol) and cholesterol ester (CE) levels were significantly reduced in the CSF of RTT patients compared to healthy controls, whereas cholesterol levels in the plasma remain unaltered in the analyzed cohort. Further, total levels of ceramides (Cer), lysophosphatidylcholine (LPC), PC-O, phosphatidylcholine (PC), were significantly reduced in the CSF of patients with RTT compared to healthy controls. Results of the statistical analysis are also listed in Table S3. In the plasma of RTT patients, we found no significant differences in lipid classes compared to healthy controls. 

**Figure 4:**
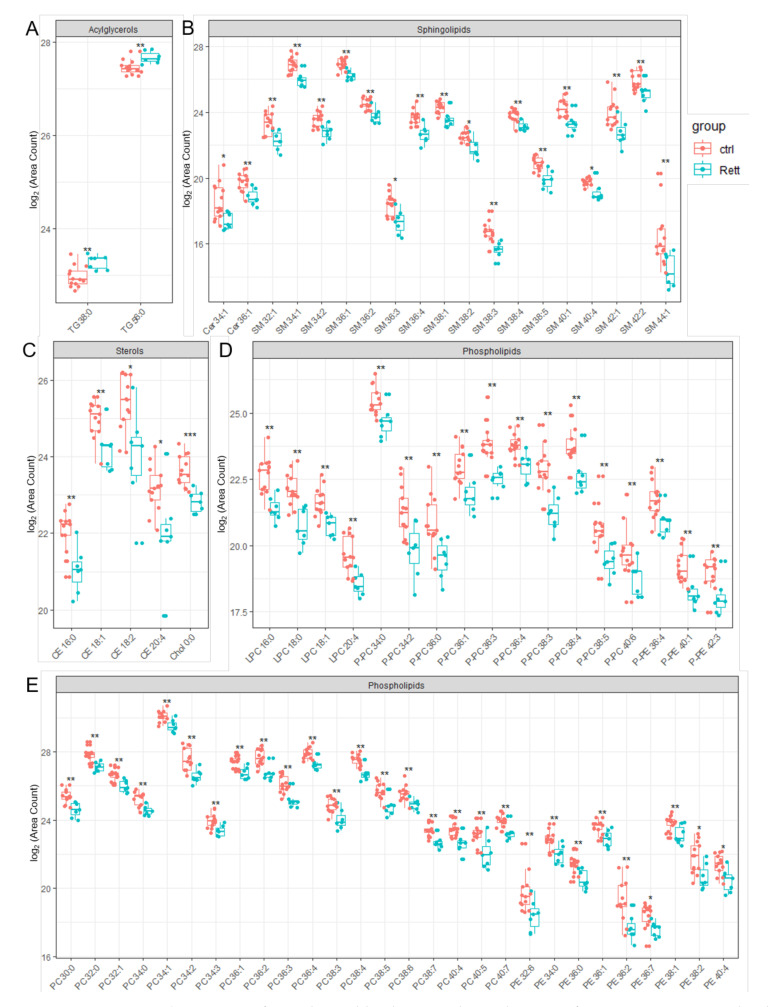
Significant changed lipid species detected in CSF of RTT patients compared to healthy controls. Data is displayed as log_2_ of total area count. (**A**) TG (Triacylglycerol), (**B**) Sphingolipids including SM (Sphingomyelin) and Cer (Ceramide), (**C**) Sterols including Chol (Cholesterol) and CE (Cholesterol Ester), (**D**,**E**) Phospholipids including LPC (Lyso- Phosphatidylcholine), LPE (Lyso-Phosphatidylserine), PC-P (Ether-linked PC), PE-P (Plasmalogens of PE), PC (Phosphatidylcholine) and PE (Phosphatidylethanolamine); *—FDR adjusted *p* < 0.05 by Wilcoxon rank-sum test , **—FDR adjusted *p* < 0.01 by Wilcoxon rank-sum test, ns-not significant.

Significantly changed lipid species identified in the CSF of RTT patients compared to healthy controls are depicted in Figure 4. Results of the statistics applied are also listed in Table S4 of the supplement. In addition to already shown reduced CSF cholesterol levels, we were able to identify reduced levels of five detected CE (CE 16:0, CE 18:1, CE 18:2, CE 20:4) and Ceramide (Cer 34:1, Cer 36:1) species in the CSF of RTT patients. Strikingly, the vast majority of SM, PE and PC species detected by our lipidomics approach were decreased in the CSF of RTT patients. Further, various ether-linked phospholipids, e.g., ether-linked PC (PC-P) and the plasmalogen of PE (PE-P), were found to be decreased in RTT patients compared to healthy controls. In addition, two TG species (TG 38:0, TG 56:0) were increased in the CSF of RTT patients compared to controls.

**Figure 5:**
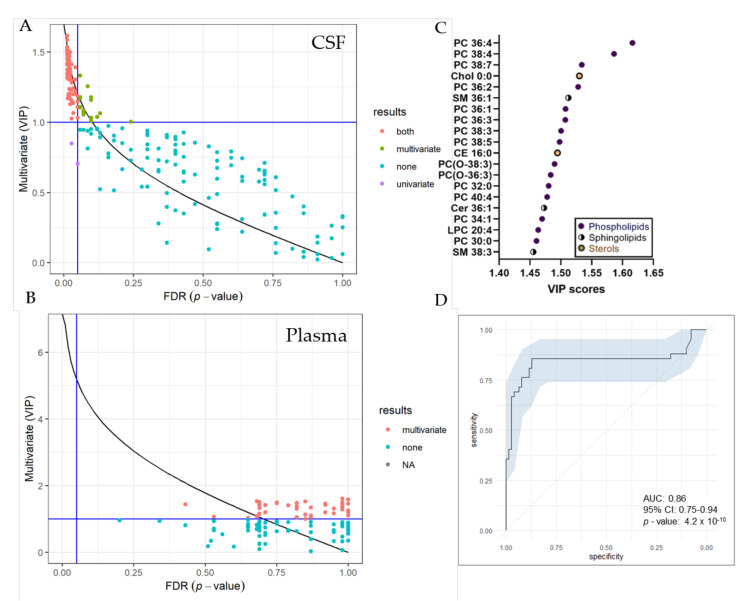
Selection of variable lipids by combined UVA and MVA approaches. (**A**,**B**) Comparison between the UVA (purple) and MVA (green) selection for CSF (**A**) and plasma (**B**) data sets. Lipids are colored according to their selection by one of the approaches or both (red). The pFDR = 0.05 (respectively, Variable Projection of Importance (VIP) = 1) threshold are displayed as a vertical (respectively, horizontal) line. Plot was created using R and the ropls package (**C**) VIP score plot of 20 most important lipid species identified in the CSF of RTT patients compared to controls. (**D**) Receiver Operating Characteristic (ROC) curve for CSF dataset, with the Area under the curve (AUC) value. The ROC curve was created from 74 significantly altered lipid species detected by both, UVA and MVA using the R packages mleval, caret and ggplot.

UVA (pFDR) and MVA (Variable Projection of Importance-VIP) metrics were compared graphically (Figure 5). In total, we identified 74 significantly changed lipid species in the CSF by both, UVA and MVA (pFDR < 0.05 and VIP > 1), highlighting sterol, as well as various phospholipid and sphingolipid species as most important variables (Figure 5C). Seventeen lipid species were only changed by MVA (VIP < 1) and two lipids only by UVA (pFDR < 0.05). In the plasma, we detected 55 significantly changed lipid species by MVA (VIP > 1), albeit we obtained no changes by UVA. A list of significantly changed lipid species and VIP scores can be found in Table S4. Area-Under-Curve (AUC)-Receiver Operating Characteristic (ROC) curve was created in order to describe the discrimination accuracy of our model and to validate the False Positive Rate (Figure 5C). An ROC curve was prepared using significantly altered lipids in the CSF detected by both, UVA and MVA (pFDR < 0.05 and VIP > 1). With a given area-under-curve (AUC)-ROC value of 0.86 (95% Confidence Interval: 0.75–0.94) in CSF samples, we are able to state a good performance of the model. 

## 3. Discussion

Previous studies on metabolic perturbations in RTT reported, that a subset of patients shows elevated levels of peripheral cholesterol, triglycerides and/or LDL upon routine diagnostic analysis [16,25]. To date, little is known about metabolomics and lipidomics perturbation occurring in the CNS of RTT patients. Previous studies, focusing on single metabolites, reported a reduction of folate, biogenic amines, pterin and serotonin levels in the CSF in a subset of RTT patients [26,27]. Ormazabal et al. showed a positive association of decreased folate levels with epilepsy, whereas the study performed by Temudo et al. did not confirm these findings. Further, folate supplementation did not improve symptoms of RTT patients. Contradicting results were also obtained concerning dopamine levels in the CSF of RTT patients, whereas a dependency of symptom severity and patient age on measurements values was suggested [28,29,30,31]. 

In this study, we describe for the first time a detailed lipidomics analysis of plasma and CSF in RTT patients. MVA, but not UVA lipidomics analysis confirmed increased DG and TG species in the plasma. As a novelty, MS revealed decreased cholesterol levels in the CSF of RTT patients. This is in contrast to studies in *Mecp2*-deficient mice, which showed an increase of cholesterol in whole brain homogenates, whereas a reduction of cholesterol precursors and decreased cholesterol synthesis rate to unaffected littermates were also reported [4,32,33]. In addition to the fact, that CSF and brain homogenates are not directly comparable, mice show a significantly higher cholesterol turnover rate compared to humans [34]. Interestingly, abnormal cholesterol and lipid parameters have also been reported in adult onset neurodegenerative diseases, such as Alzheimer’s and Parkinson’s disease [35], and may have a major impact on brain dysfunction in Smith-Lemli-Opitz Syndrome, a cholesterol synthesis defect [36]. The brain as the richest cholesterol-containing organ holds approximately 20% of all body’s cholesterol [37]. Cholesterol is particularly important for myelin formation, dendrite remodeling, neuropeptide formation, synaptogenesis, membrane trafficking and signal transduction. Cholesterol metabolism in the CNS is separate from systemic cholesterol metabolism and cholesterol is not able to cross the blood-brain barrier (BBB) [38]. Any cholesterol required in the brain must be synthesized in situ via the so-called Mevalonate pathway. The largest part of brain cholesterol resides in myelin where it integrates into the membrane bilayers, increases its rigidity and stabilizes myelin lipids and proteins [39]. Imaging studies of patients with RTT display no evidence of reduced myelin formation or myelin instability [40]. In contrast, *Mecp2*-deficient rat oligodendrocytes showed downregulation of myelin gene expression and hence an impact on myelination [41]. 

In addition to its function in myelin sheaths, cholesterol is an essential component of the membranes of astrocytes and neurons. Even though imaging studies of patients with RTT display no evidence of malformation or neurodegeneration, cell morphologic changes with smaller and more closely packed neurons with reduced dendritic complexity have been shown [42]. In *Mecp2*-deficient mice, small abnormalities in cholesterol metabolism were found to have major effects on neuronal function, but the regulatory link is yet to be elucidated [4,12]. 

Our lipidomics study demonstrates that beyond cholesterol various other membranes- and myelin-forming lipid classes and species were reduced in CSF of RTT patients. The most significant finding in lipidomics analysis was the reduction of SM in the CSF compared to healthy controls. Inline, Cappuccio et al. detected altered sphingolipid metabolism using 14 plasma samples of RTT patients carrying the *MECP2* mutation [13]. Further, Chin et al. reported decreased levels of sphingomyelin and ceramide groups in neurons derived from induced pluripotent stem cells from RTT patients [43]. SM is mainly present in cell membranes, especially in membranous myelin sheaths, where it is involved in signal transduction pathways and the regulation of cholesterol and protein trafficking to the myelin [44,45]. In addition, previous studies reported a decrease of major SM species in the CSF of Alzheimer’s disease and Multiple Sclerosis patients, suggesting SM as a potential biomarker candidate for neurodegenerative diseases [46,47,48]. Interestingly, on a molecular basis, cholesterol and sphingolipids (SM, Cer, HexCer) are the main constituents of lipid rafts, highly ordered microscopic domains of cellular membranes enabling increased activity of certain signaling pathways by biophysical stabilization of specific membrane proteins [49]. These pathways include neuronal growth, dendritic-axonal arborization, and neuronal receptor cross-talk and internalization [50]. It could be worthwhile to keep this fact in mind for further functional studies on the role of lipids in the onset of RTT.

In addition, MS analysis in the CSF of RTT patients compared to healthy controls revealed a decrease of various PL and ether-linked PL including plasmalogens. PL and their subclass, ether-linked PL, are one of the three major classes of membrane lipids in myelin sheaths and are responsible for membrane mobility, cholesterol trafficking, exo- and endocytosis due to their fusogenic ability [51]. Previous studies in mice showed that PC is able to restore neuronal plasticity and ameliorate neuronal alterations caused by inflammation [52]. Inline, previous in vitro studies investigating neurons of *Mecp2*-knockdown mice reported a crucial role of the PC synthesis pathway by enabling improved neuronal morphology [53]. Our lipidomics analysis revealed, that especially PC and PE species were reduced in the CSF of RTT patients. Deficiency of ether-PL is correlated with severe nervous system pathology and dysfunction in peroxisomal disorders [51]. In *Mecp2*-deficient mice, studies on PL levels were contradictory with Seyfried et al. reporting no change in brain PL levels [54], whereas Viola et al. found that levels of PL subclasses, PC and PE, were increased [55]. 

Previously reported metabolomics analysis on the plasma of 34 RTT patients identified metabolic pathway abnormalities in oxidative stress, mitochondrial dysfunction, and alterations in the gut microbiome compared to their 37 unaffected age- and gender-matched siblings [56]. In *Mecp2*-deficient mice, metabolic fingerprinting of the cortex showed affected amino acid, carbohydrate, as well as lipid metabolism and altered levels of several neurotransmitters [57]. In this study, MVA did not find a clear separation of plasma and CSF of MS data generated in positive ion mode of the RTT study cohort and the control group and we, therefore, did not pursue further metabolomics analysis. 

To date diagnosis for RTT is based on proof of pathogenic variants in the regulatory gene function of *MECP2* [58]. According to the RettBase, established for the collection of RTT-genomic variants, 925 different *MECP2* variants, including 215 variants of unknown significance (VUS) have been described [18]. Lipidomics profiling in RTT needs further validation but has the potential to serve as a biomarker for RTT, which may be helpful in the interpretation of VUS and become even more important in the development of therapeutic options and as a surrogate parameter for treatment monitoring. 

## 4. Material and Methods

### 4.1. Study Design

This study was conducted as part of a phase I clinical study based at the Department of Neurology, University Hospital Basel, Switzerland [24]. The main goal of this study (FINGORETT) was to assess the safety and efficacy of oral Fingolimod (FTY720, Gilenya^®^, Novartis, Basel, Switzerland) in children with RTT aged above six years along with CSF and MRI investigations. This study design opened the opportunity for a sub-study located at the Medical University of Graz by employing both, targeted and untargeted UHPLC-MS/MS of the metabolome and lipidome in plasma and CSF. In collaboration with the Department of Pediatric Neurology, Klinikum Kassel, Germany and the Department of Pediatrics, Medical University of Innsbruck, Austria we were able to increase the sample size of CSF and plasma samples obtained from RTT patients and healthy controls. Clinical parameters of patients enrolled in the study are listed in Table S1.

### 4.2. Collection of Medical Information and Specimens from Patients

Patients and/or their legal guardians were asked for informed consent prior to blood collection and/or the spinal tap performed according to the study protocol of the main study. The remaining samples were taken after informed consent in the context of routine diagnostic procedures which included 0.5–1.0 mL of additional CSF and 1.0 mL of whole blood for plasma isolation (in lithium heparin tubes). The study population involved a control group of healthy children and one study group. CSF and plasma samples were stored at −80 °C prior to analysis.

### 4.3. Chemicals

All chemicals used for sample preparation (metabolite and lipid extraction) were purchased from Merck (Darmstadt, Germany).

### 4.4. Metabolite and Lipid Extraction from CSF and Plasma Samples

For metabolomics analysis, a 3:1 volume of ice-cold ACN/MeOH/acetone (1/1/1) was added to 50 µL of plasma and 100 µL of CSF and vortexed for 15 s as described previously [59,60]. The sample was then allowed to precipitate at 4 °C for 60 min, followed by centrifugation at 12,000 rpm, for 10 min. The resultant supernatant was transferred into a clean Eppendorf tube and concentrated under a stream of dry nitrogen gas at room temperature. The resultant plasma and CSF sample pellet were resuspended in ACN/water 1:1 (*v/v*) to the original and 0.5 sample volume, respectively, and subjected to MS analysis or immediately placed at −80 °C until further analysis.

For lipidomics analysis, the liquid extraction protocol with methyl-tert-butyl ether (MTBE) was applied [61]. For this, 1.5 mL methanol and 5 mL MTBE were added to 50 µL of plasma or to 150 µL of CSF in 12 mL glass test tubes with PTFE-lined caps; 10 µL of 0.5 mM PE 24:0 was added as extraction control. The mixture was incubated for 10 min in an overhead shaker at room temperature. After the addition of 1.25 mL deionized water and 10 min of additional shaking, the mixture was centrifuged for 5 min at 2000× *g* and the upper phase was transferred to a new test tube. The lower aqueous phase was re-extracted with 2 mL of the upper phase of MTBE/methanol/deionized water 10:3:2.5 (*v*/*v*/*v*). The upper phases were combined, evaporated in a vacuum centrifuge and dissolved in 500 µL chloroform/methanol 1:1 (*v*/*v*) for storage at −80 °C. For MS analysis of the lipidome, plasma and CSF samples were evaporated under a stream of nitrogen and the resultant pellet was resuspended in the original or 0.5 sample volume, respectively, of isopropanol/methanol/water (95:5:5). 

### 4.5. Mass Spectrometric Analysis

A full-scan mass-spectrometric analysis of each sample’s metabolite and lipid components was achieved by Dionex Ultimate XRS UHPLC (ultra-high performance liquid chromatography)-Orbitrap Velos Pro hybrid mass spectrometer (Thermo Fisher Scientific, Waltham, MA, USA) operated in Data Dependent Acquisition mode using a HESI II ion source [59,62,63]. This high resolution technique provided a comprehensive analysis of the sample’s metabolomics and lipidomics profile. Metabolomics analysis was measured in positive electrospray ionization mode and lipidomics analysis in both positive and negative electrospray ionization modes. LC-MS/MS parameters applied for metabolomics and lipidomics analysis are listed in Table 1. Full scan profile spectra were acquired in the Orbitrap mass analyzer at a resolution setting of 100,000 at *m*/z 400. For MS/MS experiments, the 10 most abundant ions of the full scan spectrum were sequentially fragmented in the ion trap using He as collision gas (Normalized Collision Energy: 50; Isolation width: 1.5; Activation Q: 0.2; Activation Time: 10) and centroided product spectra were collected. The exclusion time was set to 10 s. This protocol generated three complementary datasets for each sample collected and provided a complete and data-rich profile of the individuals’ metabolome and lipidome at the given time point. Respective pooled CSF and plasma samples were used as quality controls (QC). These QC were repeatedly measured at every 10th position in the acquisition line to examine and if necessary to correct for systematic errors.

### 4.6. Data Analysis and Statistics

All raw files obtained from high-resolution MS were either imported into the Compound discoverer 2.0 software (provided by Thermo Fisher Scientific, Waltham, MA, USA) for metabolite identification, or into the Lipid Data Analyzer (LDA) software (version 2.8.0, Graz, Austria) [64] for lipid content analysis [65]. The data set obtained were used to generate a potential target list of compounds taking into account different confidence levels in order to minimize wrong feature validation and maximize the association of features with known metabolites [59]. Special attention was paid to the presence of drug metabolites used in the context of sedation for the spinal tap and/or treatment of the underlying disease. These data points were recognized and filtered out prior to statistical analysis. PE 24:0 was used as extraction control. 

To evaluate the variance, the study and the healthy control group datasets obtained underwent statistical analysis using the programming language R (version 4.1.1) and the *lipidr* package [23]. Variables without significant variation were not considered further. Data were analyzed using the Wilcoxon rank-sum test with correction for multiple comparisons via false discovery rate (FDR). * *p* < 0.05, ** *p* < 0.01, *** *p* < 0.001

## 5. Conclusions

Taking into account that cholesterol, various PL species including plasmalogens and SM are decreased in the CSF of RTT patients we hypothesize, that the composition of membrane lipids is impaired and is a major cause of CNS dysfunction in RTT. Future functional studies are aiming to address this issue. In contrast to CSF data, MVA of plasma samples identified various PL and acylglycerols as major separators between the RTT patient and healthy control group, whereas UVA did not detect significant lipids in the patient group. In conclusion, this study provides insights into changes of the lipidome occurring in the CSF of RTT patients. In view of emerging therapies, this study may provide a basis for future biomarker discovery that may help in the delineation of VUS in MeCP2 and serve as surrogate parameters to study treatment effects. 

## Figures and Tables

**Table 1:** LC-MS/MS Parameters used for the Metabolomics and Lipidomics Analysis.

	Metabolomics Method	Lipidomics Method
Column	Acquity UPLC BEH Amide, 2.1 mm × 150 mm, 1.7 µm (Waters Corporation, Milford, MA, USA)	Acquity UPLC BEH C8 column, 1 mm × 100 mm, 1.7 µm (Waters Corporation, Milford, MA, USA)
Mobile Phase A	97% ACN + 3% H_2_O + 0.1 mM NH_4_COOH + 0.16% HCOOH	H_2_O + 0.1 mM NH_4_COOH + 0.16% HCOOH
Mobile Phase B	H_2_O + 0.1 mM NH_4_COOH + 0.16% HCOOH	ACN/IPA (5:2, *v*/*v*) + 0.1 mM NH_4_COOH + 0.16% HCOOH
Gradient	Gradient elution started at 5% mobile phase B and increased up to 30% over 30 min. Mobile phase B was reset to start conditions over a minute and re-equilibrated for 9 min	Gradient elution started at 50% mobile phase B, rising to 100% B over 40 min; 100% B was held for 10 min and the column was re-equilibrated with 50% B for 8 min
Injection volume	2 µL	2 µL
Flow Rate	200 µL min^−1^	150 µL min^−1^
Separation temperature	50 °C	40 °C
Autosampler temperature	8°C	8°C
Ion Source Parameters		
Source Voltage	3.8 kV	4.5 kV (positive ion mode) 3.8 kV (negative ion mode)
Source Temperature	250 °C	275 °C (positive ion mode) 325 °C (negative ion mode)
Sheath Gas	40 AU	25 AU (positive ion mode) 30 AU (negative ion mode)
AUX Gas	9 AU	9 AU (positive ion mode) 10 AU (negative ion mode)
Sweep Gas	0 AU	0 AU
Capillary Temperature	300 °C	300 °C
Acquisition of Full-Scan Spectra	*m*/*z* 60–1600	*m*/*z* 400–1200 (positive ion mode) *m*/*z* 400–1600 (negative ion mode)

## Data Availability

The data presented in this study are openly available in zenodo at https://doi.org/10.5281/zenodo.5947668. Raw data are provided in Panasonic Raw Image format, data processed with LDA is available in excel files. An additional excel file named “metadata” provides sample information.

## Supplementary Materials

The following supporting information can be downloaded at: https://www.mdpi.com/article/10.3390/metabo12040291/s1, Figure S1: PCA Plot, Permutation Test and Observation Diagnostics. Table S1: Clinical Parameters of RTT Patient Study Cohort, Table S2: Loading Plot of CSF Samples, Table S3: Loading Plot of Plasma Samples, Table S4: Wilcoxon Rank-Sum Test of Lipid Classes Detected in CSF Samples, Table S5: VIP Plot of CSF Data.
