# Supplementary material for: Changes in the Cerebrospinal Fluid and Plasma Lipidome in Patients with Rett Syndrome
Source: Metabolites. 2022 Mar 25;12(4):291. doi: 10.3390/metabo12040291 (PMC9026385; doi:10.3390/metabo12040291)
Supplement: Supplementary file 1 [file metabolites-12-00291-s001.zip › metabolites-1614169-supplementary.pdf]

metabolites

MDPI

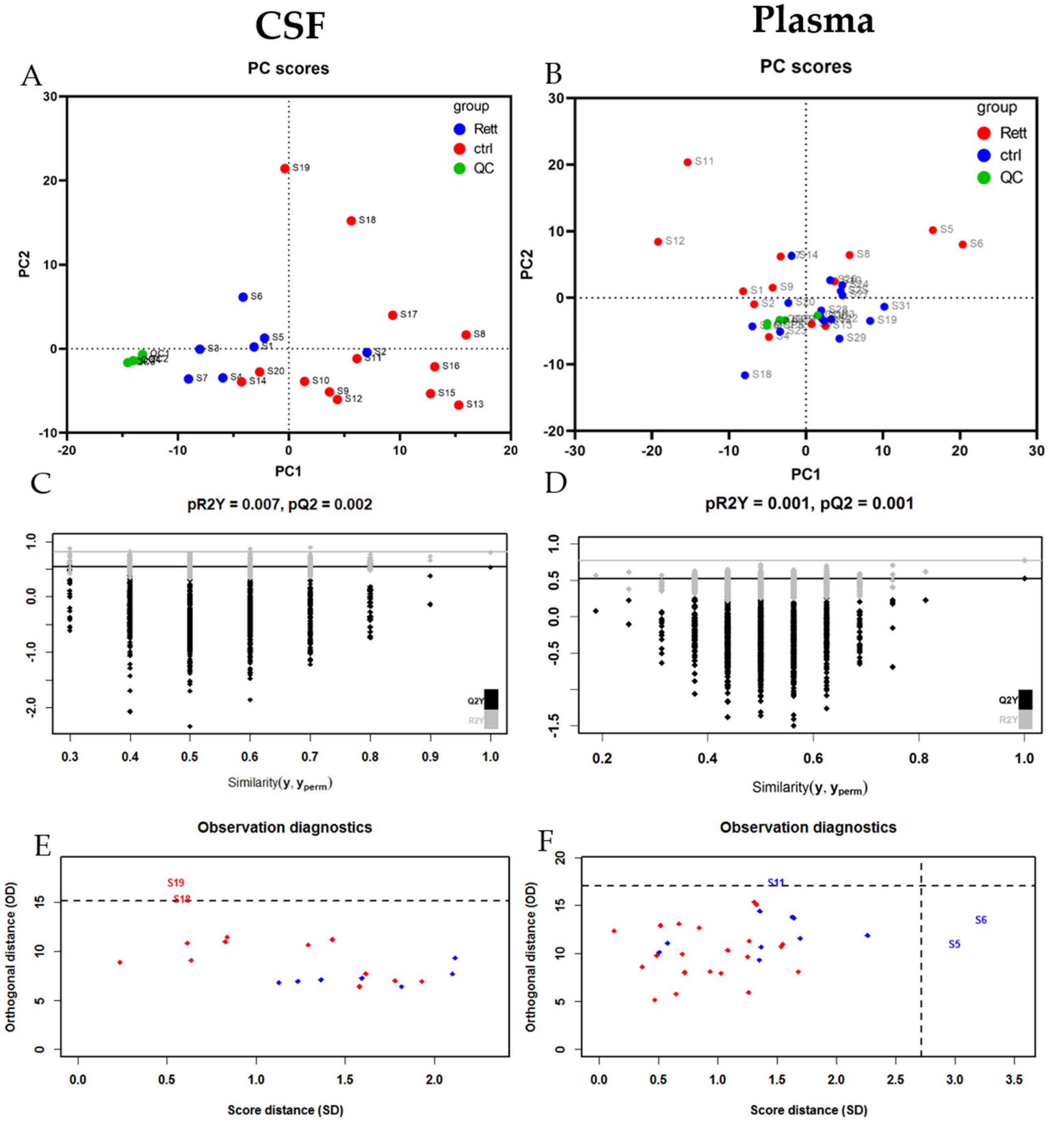

Figure S1. PCA plot (**A** for CSF, **B** for plasma), Permutation Test (with 1000 random permutations) (**C** for CSF, **D** for plasma) and Observation Diagnostics Identifying Potential Outliers (**E** for CSF, **F** for plasma).

Table S1. Clinical Parameters of RTT Patient Study Cohort.

	**All**	**Individual Patients**													
**Number of Patients**	13	001	002	003	004	005	006	007	008	009	010	011	012	013	014
**Number of Females (%)**	100														
**Sample Type CSF (C)/Plasma (P)**		C/P	C/P	C/P	C/P	C/P	C/P	C	P	P	P	P	P	P	P
**Age (mean and SD) in Years**	8.4 (3.6)	12	13	12	10	6	15	5	9	3	6	9	5	8	5
***MECP2* Mutation**	100%	+	+	+	+	+	+	+	+	+	+	+	+	+	+
**Rett Stage (Hagberg)**		III	III	III	IV	III	II	III	III	III	III	III	III	III	III
**Seizures (%)**	57.1%	+	+	-	+	+	-	+	+	-	+	+	-	-	-
**MRI**		N or	M od	Mi cr	N or	De ce	N or	At ro	no t	N or	N or	N or	N or	N or	N or

Table S2. Loading Plot of CSF Samples.

molrank	Molecule	Class	chain1
1	PC 36:4	PC	36:4
2	PC 38:4	PC	38:4
3	PC 38:7	PC	38:7
4	Chol 0:0	Chol	0:0
5	PC 36:2	PC	36:2
6	SM 36:1	SM	36:1
7	PC 36:1	PC	36:1
8	PC 36:3	PC	36:3
9	PC 38:3	PC	38:3
10	PC 38:5	PC	38:5
11	CE 16:0	CE	16:0
12	PC(O-38:3)	PC	38:3
13	PC(O-36:3)	PC	36:3
14	PC 32:0	PC	32:0
15	PC 40:4	PC	40:4
16	Cer 36:1	Cer	36:1
17	PC 34:1	PC	34:1
18	LPC 20:4	LPC	20:4
19	PC 30:0	PC	30:0
20	SM 38:3	SM	38:3
21	PC 34:0	PC	34:0
22	PC 32:1	PC	32:1
23	CE 18:1	CE	18:1
24	PC 40:7	PC	40:7
25	PE(P-40:1)	PE	40:1
26	SM 38:1	SM	38:1
27	SM 34:1	SM	34:1
28	LPC 16:0	LPC	16:0
29	SM 36:2	SM	36:2
30	PC 40:5	PC	40:5
31	LPC 18:0	LPC	18:0
32	PC 34:2	PC	34:2
33	SM 38:5	SM	38:5
34	SM 38:4	SM	38:4
35	PC(O-36:4)	PC	36:4

36	PE 40:4	PE	40:4
37	PE 36:1	PE	36:1
38	PE 34:0	PE	34:0
39	SM 40:1	SM	40:1
40	PE 38:1	PE	38:1
41	SM 32:1	SM	32:1
42	PC 34:3	PC	34:3
43	SM 36:4	SM	36:4
44	PE 36:0	PE	36:0
45	PE(P-42:9)	PE	42:9
46	PC(O-38:4	PC	38:4
47	SM 34:2	SM	34:2
48	LPC 18:1	LPC	18:1
49	SM 42:1	SM	42:1
50	PC(O-34:2)	PC	34:2

Table S3. Loading Plot of Plasma Samples.

molrank	Molecule	Class	chain1
1	LPE 22:0	LPE	22:0
2	PS 40:1	PS	40:1
3	TG 54:7	TG	54:7
4	TG 54:6	TG	54:6
5	LPE 18:1	LPE	18:1
6	TG 52:5	TG	52:5
7	CE 20:1	CE	20:1
8	LPE 20:1	LPE	20:1
9	PC(O-38:3)	PC	38:3
10	DG 36:4	DG	36:4
11	PC 36:5	PC	36:5
12	PE 40:2	PE	40:2
13	TG 58:9	TG	58:9
14	TG 54:5	TG	54:5
15	LPE 20:0	LPE	20:0
16	TG 56:5	TG	56:5
17	TG 56:8	TG	56:8
18	PS 40:5	PS	40:5
19	CE 20:2	CE	20:2
20	TG 56:7	TG	56:7
21	LPI 18:1	LPI	18:1
22	DG 34:2	DG	34:2
23	LPI 18:2	LPI	18:2
24	PI 36:3	PI	36:3
25	TG 58:8	TG	58:8
26	LPC 18:2	LPC	18:2
27	PE 40:6	PE	40:6
28	PE 36:3	PE	36:3
29	DG 32:1	DG	32:1
30	PI 38:6	PI	38:6
31	PC 40:6	PC	40:6
32	PS 26:2	PS	26:2
33	DG 36:1	DG	36:1
34	PC(O-40:4)	PC	40:4
35	TG 52:4	TG	52:4

36	PS 40:3	PS	40:3
37	PC 40:7	PC	40:7
38	PE 40:1	PE	40:1
39	LPC 20:3	LPC	20:3
40	LPC 16:1	LPC	16:1
41	TG 54:4	TG	54:4
42	PS 38:4	PS	38:4
43	PI 38:5	PI	38:5
44	PS 38:1	PS	38:1
45	DG 36:3	DG	36:3
46	LPC 18:1	LPC	18:1
47	DG 36:2	DG	36:2
48	PC(O-42:4)	PC	42:4
49	DG 32:0	DG	32:0
50	LPC 20:4	LPC	20:4

Table S4. Wilcoxon Rank-Sum Test of Lipid Classes Detected in CSF Samples.

	class	group1	group2	n1	n2	p	p(FDR)	p.signif	method
1	CE	ctrl	Rett	13	7	0.01450	0.02900	*	Wilcoxon
2	Cer	ctrl	Rett	13	7	0.00846	0.01970	*	Wilcoxon
3	Chol	ctrl	Rett	13	7	0.000181	0.00253	**	Wilcoxon
4	DG	ctrl	Rett	13	7	0.485000	0.52230	ns	Wilcoxon
5	LPC	ctrl	Rett	13	7	0.001700	0.00793	**	Wilcoxon
6	LPS	ctrl	Rett	13	7	0.067500	0.10500	ns	Wilcoxon
7	PC-O	ctrl	Rett	13	7	0.004670	0.01307	*	Wilcoxon
8	PE-P	ctrl	Rett	13	7	0.135000	0.18900	ns	Wilcoxon
9	PC	ctrl	Rett	13	7	0.001700	0.00793	**	Wilcoxon
10	PE	ctrl	Rett	13	7	0.018600	0.03255	*	Wilcoxon
11	PI	ctrl	Rett	13	7	0.27500	0.32083	ns	Wilcoxon
12	PS	ctrl	Rett	13	7	0.27500	0.32083	ns	Wilcoxon
13	SM	ctrl	Rett	13	7	0.002430	0.00850	**	Wilcoxon
14	TG	ctrl	Rett	13	7	0.53600	0.53600	ns	Wilcoxon

Table S5. VIP Plot of CSF Data.

	lipid	group1	group2	p.adj	p	p.signif	method	vipVn	pvaVn	results
**1**	PC 36:4	ctrl	Rett	0.012	0.00116	**	Wilcoxon	1.61605385119425	0.012	both
**2**	PC 38:4	ctrl	Rett	0.012	0.00116	**	Wilcoxon	1.58626736872581	0.012	both
**3**	PC 38:7	ctrl	Rett	0.012	0.00116	**	Wilcoxon	1.53388994943701	0.012	both
**4**	Chol 0:0	ctrl	Rett	0.012	0.00018	***	Wilcoxon	1.53006337846651	0.012	both
**5**	PC 36:2	ctrl	Rett	0.012	0.00116	**	Wilcoxon	1.52808444044615	0.012	both
**6**	SM 36:1	ctrl	Rett	0.012	0.00077	***	Wilcoxon	1.51234067128772	0.012	both
**7**	PC 36:1	ctrl	Rett	0.012	0.00116	**	Wilcoxon	1.50773491056356	0.012	both
**8**	PC 36:3	ctrl	Rett	0.017	0.00243	**	Wilcoxon	1.50723951188389	0.017	both

**9**	PC 38:3	ctrl	Rett	0.02	0.00467	**	Wilcoxon	1.50040111725706	0.02	both
**10**	PC 38:5	ctrl	Rett	0.023	0.00635	**	Wilcoxon	1.49800537004758	0.023	both
**11**	CE 16:0	ctrl	Rett	0.018	0.00341	**	Wilcoxon	1.49446302884178	0.018	both
**12**	PC(O-38:3)	ctrl	Rett	0.012	0.00031	***	Wilcoxon	1.48993333548825	0.012	both
**13**	PC(O-36:3)	ctrl	Rett	0.012	0.00018	***	Wilcoxon	1.48354071993397	0.012	both
**14**	PC 32:0	ctrl	Rett	0.012	0.00116	**	Wilcoxon	1.48022796089679	0.012	both
**15**	PC 40:4	ctrl	Rett	0.017	0.00243	**	Wilcoxon	1.47789817626089	0.017	both
**16**	Cer 36:1	ctrl	Rett	0.02	0.00467	**	Wilcoxon	1.4729099455919	0.02	both
**17**	PC 34:1	ctrl	Rett	0.02	0.00467	**	Wilcoxon	1.46963483314579	0.02	both
**18**	LPC 20:4	ctrl	Rett	0.018	0.00341	**	Wilcoxon	1.46337859007862	0.018	both
**19**	PC 30:0	ctrl	Rett	0.017	0.00243	**	Wilcoxon	1.46072840991137	0.017	both
**20**	SM 38:3	ctrl	Rett	0.012	0.00077	***	Wilcoxon	1.45554866570694	0.012	both
**21**	PC 34:0	ctrl	Rett	0.014	0.0017	**	Wilcoxon	1.44615765257223	0.014	both
**22**	PC 32:1	ctrl	Rett	0.014	0.0017	**	Wilcoxon	1.44200523679701	0.014	both
**23**	CE 18:1	ctrl	Rett	0.02	0.00467	**	Wilcoxon	1.43936513631138	0.02	both
**24**	PC 40:7	ctrl	Rett	0.02	0.00467	**	Wilcoxon	1.4387747193428	0.02	both
**25**	PE(P-40:1)	ctrl	Rett	0.018	0.00341	**	Wilcoxon	1.43466530089699	0.018	both
**26**	SM 38:1	ctrl	Rett	0.018	0.00341	**	Wilcoxon	1.42589643124525	0.018	both
**27**	SM 34:1	ctrl	Rett	0.012	0.00077	***	Wilcoxon	1.41950132177897	0.012	both
**28**	LPC 16:0	ctrl	Rett	0.012	0.00049	***	Wilcoxon	1.41742350258921	0.012	both
**29**	SM 36:2	ctrl	Rett	0.012	0.00116	**	Wilcoxon	1.4142723330899	0.012	both
**30**	PC 40:5	ctrl	Rett	0.028	0.00846	**	Wilcoxon	1.41407124579304	0.028	both
**31**	LPC 18:0	ctrl	Rett	0.012	0.00077	***	Wilcoxon	1.41334518997063	0.012	both
**32**	PC 34:2	ctrl	Rett	0.012	0.00077	***	Wilcoxon	1.41209707685598	0.012	both
**33**	SM 38:5	ctrl	Rett	0.012	0.00077	***	Wilcoxon	1.4118465392347	0.012	both
**34**	SM 38:4	ctrl	Rett	0.018	0.00341	**	Wilcoxon	1.40162278168119	0.018	both
**35**	PC(O-36:4)	ctrl	Rett	0.018	0.00341	**	Wilcoxon	1.40009989416406	0.018	both

**36**	PE 40:4	ctrl	Rett	0.042	0.01447	*	Wilcoxon	1.39083285015713	0.042	both
**37**	PE 36:1	ctrl	Rett	0.023	0.00635	**	Wilcoxon	1.38263372450345	0.023	both
**38**	PE 34:0	ctrl	Rett	0.023	0.00635	**	Wilcoxon	1.37734257185986	0.023	both
**39**	SM 40:1	ctrl	Rett	0.012	0.00116	**	Wilcoxon	1.37385702293612	0.012	both
**40**	PE 38:1	ctrl	Rett	0.023	0.00635	**	Wilcoxon	1.36669345206384	0.023	both
**41**	SM 32:1	ctrl	Rett	0.023	0.00635	**	Wilcoxon	1.34921850374917	0.023	both
**42**	PC 34:3	ctrl	Rett	0.018	0.00341	**	Wilcoxon	1.34822385689356	0.018	both
**43**	SM 36:4	ctrl	Rett	0.012	0.00049	***	Wilcoxon	1.34546885675748	0.012	both
**44**	PE 36:0	ctrl	Rett	0.014	0.0017	**	Wilcoxon	1.34116704606902	0.014	both
**45**	PC(O-38:4)	ctrl	Rett	0.023	0.00635	**	Wilcoxon	1.33171378338387	0.023	both
**46**	SM 34:2	ctrl	Rett	0.014	0.0017	**	Wilcoxon	1.32988345622569	0.014	both
**47**	LPC 18:1	ctrl	Rett	0.012	0.00077	***	Wilcoxon	1.31470008829537	0.012	both
**48**	SM 42:1	ctrl	Rett	0.017	0.00243	**	Wilcoxon	1.30627767791707	0.017	both
**49**	PC(O-34:2)	ctrl	Rett	0.023	0.00635	**	Wilcoxon	1.30608480699148	0.023	both
**50**	PE 38:2	ctrl	Rett	0.042	0.01447	*	Wilcoxon	1.30475472336541	0.042	both
**51**	SM 36:3	ctrl	Rett	0.028	0.00846	**	Wilcoxon	1.2997977895814	0.028	both
**52**	PE 36:2	ctrl	Rett	0.028	0.00846	**	Wilcoxon	1.28276173305773	0.028	both
**53**	PE 36:7	ctrl	Rett	0.034	0.01115	*	Wilcoxon	1.24235987053954	0.034	both
**54**	PC(O-34:0)	ctrl	Rett	0.02	0.00467	**	Wilcoxon	1.23653127672912	0.02	both
**55**	PE(P-42:3)	ctrl	Rett	0.028	0.00846	**	Wilcoxon	1.22869826466978	0.028	both
**56**	SM 40:4	ctrl	Rett	0.042	0.01447	*	Wilcoxon	1.21858942125675	0.042	both
**57**	PC(O-36:1)	ctrl	Rett	0.02	0.00467	**	Wilcoxon	1.20339346996032	0.02	both
**58**	SM 38:2	ctrl	Rett	0.042	0.01447	*	Wilcoxon	1.20311542585463	0.042	both
**59**	PC(O-36:0)	ctrl	Rett	0.017	0.00243	**	Wilcoxon	1.20127485927774	0.017	both
**60**	CE 18:2	ctrl	Rett	0.034	0.01115	*	Wilcoxon	1.19912915707104	0.034	both
**61**	PC 38:6	ctrl	Rett	0.018	0.00341	**	Wilcoxon	1.1949949387061	0.018	both
**62**	SM 44:1	ctrl	Rett	0.02	0.00467	**	Wilcoxon	1.18577712944291	0.02	both

**63**	SM 32:2	ctrl	Rett	0.05	0.0186	*	Wilcoxon	1.18428378425499	0.05	both
**64**	TG 56:0	ctrl	Rett	0.018	0.00341	**	Wilcoxon	1.18191845803096	0.018	both
**65**	PC(O-38:5)	ctrl	Rett	0.014	0.0017	**	Wilcoxon	1.1714583910567	0.014	both
**66**	SM 42:2	ctrl	Rett	0.023	0.00635	**	Wilcoxon	1.16589352177118	0.023	both
**67**	PE 32:6	ctrl	Rett	0.028	0.00846	**	Wilcoxon	1.15488511744128	0.028	both
**68**	PC(O-34:4)	ctrl	Rett	0.05	0.0186	*	Wilcoxon	1.14939992239353	0.05	both
**69**	CE 20:4	ctrl	Rett	0.034	0.01115	*	Wilcoxon	1.13578604530624	0.034	both
**70**	PE(P-36:4)	ctrl	Rett	0.023	0.00635	**	Wilcoxon	1.12822594065193	0.023	both
**71**	PC 40:6	ctrl	Rett	0.05	0.0186	*	Wilcoxon	1.10765728786774	0.05	both
**72**	Cer 34:1	ctrl	Rett	0.034	0.01115	*	Wilcoxon	1.06607496665876	0.034	both
**73**	PE(P-38:4)	ctrl	Rett	0.05	0.0186	*	Wilcoxon	1.03177664499349	0.05	both
**74**	PC(O-40:6)	ctrl	Rett	0.028	0.00846	**	Wilcoxon	1.02534373480807	0.028	both
**75**	TG 38:0	ctrl	Rett	0.028	0.00846	**	Wilcoxon	0.849749958248299	0.028	univariate
**76**	DG 36:0	ctrl	Rett	0.05	0.0186	*	Wilcoxon	0.705671692154006	0.05	univariate
**77**	PE(P-42:9)	ctrl	Rett	0.059	0.02361	*	Wilcoxon	1.33218443833762	0.059	multivariate
**78**	PE 40:3	ctrl	Rett	0.086	0.03695	*	Wilcoxon	1.25520049272695	0.086	multivariate
**79**	LPS 24:1	ctrl	Rett	0.098	0.04556	*	Wilcoxon	1.17884833368953	0.098	multivariate
**80**	SM 44:4	ctrl	Rett	0.059	0.02361	*	Wilcoxon	1.17883139625663	0.059	multivariate
**81**	PC 36:5	ctrl	Rett	0.059	0.02361	*	Wilcoxon	1.1713056482212	0.059	multivariate
**82**	PE 40:2	ctrl	Rett	0.098	0.04556	*	Wilcoxon	1.15862414558351	0.098	multivariate
**83**	LPS 22:1	ctrl	Rett	0.059	0.02361	*	Wilcoxon	1.10873604809688	0.059	multivariate
**84**	SM 40:2	ctrl	Rett	0.059	0.02361	*	Wilcoxon	1.10572413182355	0.059	multivariate
**85**	SM 42:3	ctrl	Rett	0.071	0.0297	*	Wilcoxon	1.0819764941829	0.071	multivariate
**86**	SM 44:2	ctrl	Rett	0.071	0.0297	*	Wilcoxon	1.06583415622991	0.071	multivariate
**87**	Cer 42:1	ctrl	Rett	0.13	0.06749	ns	Wilcoxon	1.06312365265887	0.13	multivariate
**88**	Cer 40:1	ctrl	Rett	0.071	0.0297	*	Wilcoxon	1.05530634992783	0.071	multivariate
**89**	LPS 26:2	ctrl	Rett	0.12	0.05568	ns	Wilcoxon	1.03830046438056	0.12	multivariate

**90**	CE 22:4	ctrl	Rett	0.098	0.04556	*	Wilcoxon	1.03336446762705	0.098	multivariate
**91**	SM 44:3	ctrl	Rett	0.098	0.04556	*	Wilcoxon	1.02610654198856	0.098	multivariate
**92**	SM 40:5	ctrl	Rett	0.098	0.04556	*	Wilcoxon	1.00809149772645	0.098	multivariate
**93**	TG 56:6	ctrl	Rett	0.24	0.1348	ns	Wilcoxon	1.00395821152119	0.24	multivariate
